# Critical appraisal of the accuracy of Acuros-XB and Anisotropic Analytical Algorithm compared to measurement and calculations with the compass system in the delivery of RapidArc clinical plans

**DOI:** 10.1186/1748-717X-8-140

**Published:** 2013-06-11

**Authors:** Murugesan Kathirvel, Shanmuga Subramanian, Alessandro Clivio, Gandhi Arun, Antonella Fogliata, Giorgia Nicolini, Vellaiyan Subramani, Shanmugam Thirumalai Swamy, Eugenio Vanetti, Luca Cozzi

**Affiliations:** 1 Yashoda Super Speciality Hospital, Hyderabad, India; 2 Department of Medical Physics, Oncology Institute of Southern Switzerland, Bellinzona, Switzerland; 3 All Indian Institute of Medical Sciences, New Delhi, India; 4 Research and Development Centre, Bharathiar University, Coimbatore, India; 5 Oncology Institute of Southern, 6504, Bellinzona, Switzerland

**Keywords:** Acuros-XB, Anisotropic Analytical Algorithm, RapidArc, Compass

## Abstract

**Background:**

The accuracy of the two dose calculation engines available for RapidArc planning (both released for clinical use) is investigated in comparison to the COMPASS data.

**Methods:**

Two dose calculation algorithms (Acuros-XB and Anisotropic Analytic Algorithm (AAA)) were used to calculate RA plans and compared to calculations with the Collapsed Cone Convolution algorithm (CC) from the COMPASS system (IBA Dosimetry). CC calculations, performed on patient data, are based on experimental fluence measurements with a 2D array of ion chambers mounted on the linac head. The study was conducted on clinical cases treated with RA. Five cases for each of the following groups were included: Brain, Head and Neck, Thorax, Pelvis and stereotactic body radiation therapy for hypo-fractionated treatments with small fields. COMPASS measurements were performed with the iMatrixx-2D array. RapidArc plans were optimized for delivery using 6MV photons from a Clinac-iX (Varian, Palo Alto, USA).

Accuracy of the RA calculation was appraised by means of: 1) comparison of Dose Volume histograms (DVH) metrics; 2) analysis of differential dose distributions and determination of mean dose differences per organ; 3) 3D gamma analysis with distance-to-agreement and dose difference thresholds set to 3%/3 mm or 2%/2 mm for targets, organs at risks and for the volumes encompassed by the 50 and 10% isodoses.

**Results:**

For almost all parameters, the better agreement was between Acuros-XB and COMPASS independently from the anatomical site and fractionation. The same result was obtained from the mean dose difference per organ with Acuros-CC average differences below 0.5% while for AAA-CC data, average deviations exceeded 0.5% and in the case of the pelvis 1%. Relevance of observed differences determined with the 3D gamma analysis resulted in a pass rate exceeding 99.5% for Acuros-CC and exceeding 97.5% for AAA-CC.

**Conclusions:**

This study demonstrated that i) a good agreement exists between COMPASS-CC calculations based on measured fluences with respect to dose distributions obtained with both Acuros-XB and AAA algorithms; ii) 3D dose distributions reconstructed from actual delivery coincide very precisely with the planned data; iii) a slight preference in favor of Acuros-XB was observed suggesting the preference for this algorithm in clinical applications.

## Background

Volumetric Modulated Arc Therapy (VMAT) is, in the variety of radiation treatment modalities, a possibly valuable but also challenging technique because of its intrinsic complexity involving advanced inverse planning algorithms, dose calculation engines applied to complex fields and dynamic delivery with several variable parameters (speed of multileaf collimator, dose rate, gantry rotational speed). All elements are highly interconnected and contribute together to the generation of dose distributions of virtually any complexity. As for all advanced treatment techniques, one fundamental aspect to monitor and to guarantee is the consistence between planning and delivery. This to prevent the risk of un-intended mistreatments with potentially severe implications for patients. The present study aims to contribute to the determination of this accuracy in a clinical environment. The VMAT model investigated here is the RapidArc solution (RA, Varian Medical Systems, USA) derived from the original prototype of Otto [1]. Several studies appraised the subject of quality assurance of delivery vs calculation of RA [2-13]. These used for benchmark of the calculations, experimental measurements with a plethora of different detectors and established consolidated practice in the clinical routine. As a general summary, all these studies suggested the safe and reliable consistency of calculations vs delivery either in simple geometrical or anthropomorphic phantoms. Some studies [14,15] addressed the usage of Monte Carlo methods to convert delivery information registered by the linacs during irradiation in input data for some sort of ”actual” in-patient dose calculation to compare to planned dose distributions. Limit of this branch of investigations is the need of computational tools not commercially available and not easily implementable in routine settings. On the same line, little has been done so far, to use measured data to recalculate the ‘actual’ dose in the patients. Investigations based on the usage of electronic portal imaging devices, used to measure transmitted dose through the patients represents the current cutting edge of the research. Pioneering studies demonstrated the possibility to reconstruct reliable dose in a quasi in-vivo setting by using as patient model the Cone Beam CT data that can be acquired daily before treatment [16]. From a different perspective, it is possible also to use measurement devices to detect the fluence generated by the delivery process before entering the patient and from this, to determine the ”actual” delivered dose inside a patient model. The COMPASS system (IBA Dosimetry, Germany) is a commercial system which allows to investigate this area. In fact, the COMPASS consists of an experimental device, the Matrix 2D array of ionization chambers which, mounted on the linac head, can measure the output fluence of any given field. This measured fluence can be used as input to a calculation engine based on a Collapsed Cone algorithm (CC) which allows to compute a 3D dose distribution in a phantom or in a patient CT dataset. In this way, although depending on the CC algorithm and the CT set used, it is possible to generate a kind of ‘’delivered” dose distribution. The COMPASS system has been studied in terms of its intrinsic accuracy compared to other measurement devices as well as in terms of its clinical usability [17-21] for IMRT techniques. In this study, COMPASS usage will be extended to VMAT 3D quality assurance.

Aim of the study is the investigation of the accuracy of the two dose calculation engines available for RA planning (the Acuros-XB and the Anisotropic Analytical Algorithm both released for clinical use) in comparison to the COMPASS data for a number of cases representing a wide spectrum of possible clinical conditions.

## Methods

### Patients’ selection

The study was designed to explore a wide range of clinical applications of RapidArc. For this reason five localisations were identified and for each of them, five patients were selected from the clinical database. Localisations (or groups) were: brain, head and neck (HN), thorax, pelvis; these represented conventional fractionation regimens and fields of medium to large size. A fifth group was defined including patients treated for stereotactic body radio-therapy (SBRT) with hypofractionated regimen and usage of small fields to define the arcs. To increase the variability of the cases, patients with different dose prescriptions were included in the study and, to make them comparable, analysis has been performed in terms of percentage doses. Tables 1, 2, 3, 4 and 5 report also the mean prescribed doses and ranges for each of the five groups. For all patients, the planning CT and structures were shared in DICOM format between the planning system and the experimental COMPASS system described below. For each patient the planning target volume (PTV) and several organs at risk were considered. These depend upon the localization and included: brain stem, chiasm, lenses, optic nerves, retina, spinal cord, parotids, oral cavity, larynx, heart, ipsi- and contra-lateral lungs, ribs, liver, rectum, bladder, bowels, femoral heads. Tables 1, 2, 3, 4 and 5 report the volumes of each of these targets and organs at risk.

**Table 1:** Brain (Prescription 46.4 Gy [45.0.-50.4])

**Parameter**	**Acuros XB**	**AAA**	**CC**	**p**
**PTV** ( Volume [cm^3^] = 446.4 ± 235.2)				
Mean [%]	100.3 ± 0.3	101.8 ± 1.0	100.9 ± 0.7	a,c
D_5%_- D_95%_ [%]	7.7 ± 1.6	7.6 ± 1.7	7.8 ± 1.7	
V_95%_ [%]	95.9 ± 1.3	97.3 ± 2.4	96.3 ± 2.0	c
V_105%_ [%]	0.8 ± 0.6	6.5 ± 3.6	2.5 ± 2.5	a,c
CI_90%_	1.2 ± 0.1	1.2 ± 0.1	1.1 ± 0.1	
**Brain Stem** ( Volume [cm^3^] = 24.2 ± 3.9)				
D_1%_ [%]	102.7 ± 2.2	104.0 ± 2.1	103.1 ± 2.7	a
D_1.8cm3_ [%]	94.6 ± 16.7	95.6 ± 17.1	95.2 ± 17.0	a
V_50Gy_ [%]	5.0 ± 11.1	5.7 ± 12.8	5.3 ± 11.9	
**Chiasm** ( Volume [cm^3^] = 1.1 ± 0.4)				
D_1%_ [%]	95.2 ± 16.1	96.6 ± 15.4	95.7 ± 16.7	
D_1.8cm3_ [%]	69.5 ± 31.6	69.3 ± 30.6	68.9 ± 30.7	
V_50Gy_ [%]	11.5 ± 25.7	14.4 ± 32.2	15.1 ± 33.8	
**Lens** ( Volume [cm^3^] = 0.3 ± 0.1)				
Mean [%]	8.8 ± 1.9	9.4 ± 2.4	8.8 ± 2.3	c
D_1%_ [%]	10.5 ± 2.3	10.9 ± 2.5	10.3 ± 2.7	c
D_1.8cm3_ [%]	7.5 ± 1.9	8.1 ± 2.3	7.4 ± 2.1	c
**Optic Nerve** ( Volume [cm^3^] = 1.2 ± 0.7)				
D_1%_ [%]	77.1 ± 28.6	78.1 ± 28.6	76.6 ± 28.7	a
D_1.8cm3_ [%]	25.8 ± 11.6	26.6 ± 12.3	26.1 ± 12.2	
**Retina** ( Volume [cm^3^] = 17.7 ± 1.5)				
Mean [%]	19.5 ± 9.7	20.2 ± 9.9	19.7 ± 9.8	a,c
D_1%_ [%]	48.9 ± 21.7	51.3 ± 21.9	50.2 ± 21.2	c
D_1.8cm3_ [%]	34.6 ± 19.1	35.5 ± 19.5	34.9 ± 18.6	a

a Acuros vs AAA, b Acuros vs CC, c AAA vs CC.

**Table 2:** **HN** (**Prescription 63**.**2 Gy** [**50**.**0**.-**70**.**5**])

**Parameter**	**Acuros XB**	**AAA**	**CC**	**p**
**PTV** ( Volume [cm^3^] = 263.4 ± 213.3)				
Mean [%]	100.2 ± 0.8	101.1 ± 1.4	100.8 ± 1.0	a,b
D_5%_- D_95%_ [%]	7.0 ± 2.6	7.1 ± 4.3	6.9 ± 3.6	
V_95%_ [%]	96.2 ± 2.0	96.8 ± 3.7	96.7 ± 3.0	
V_105%_ [%]	1.1 ± 1.9	4.4 ± 8.3	2.3 ± 4.7	
CI_95%_	1.9 ± 0.8	2.2 ± 1.1	2.0 ± 0.9	
**Spinal Cord** ( Volume [cm^3^] = 22.9 ± 4.5)				
D_1%_ [%]	61.8 ± 3.7	63.7 ± 3.5	62.5 ± 3.3	a,c
D_1.8cm3_ [%]	60.0 ± 3.7	61.7 ± 3.7	60.5 ± 3.6	a,c
**Parotids** ( Volume [cm^3^] = 38.5 ± 19.0)				
Mean [%]	48.4 ± 11.6	49.9 ± 11.4	49.0 ± 11.6	a,b,c
D_33%_ [%]	68.0 ± 21.0	69.1 ± 19.7	68.1 ± 20.1	c
D_67%_ [%]	24.6 ± 10.7	26.9 ± 11.7	26.2 ± 11.6	a
**Oral Cavity** ( Volume [cm^3^] = 76.1 ± 3.8)				
Mean [%]	66.7 ± 13.8	67.4 ± 14.1	67.3 ± 14.1	a
D_1%_ [%]	99.6 ± 1.2	99.0 ± 1.4	99.6 ± 0.9	
D_33%_ [%]	76.0 ± 12.1	76.7 ± 12.5	76.5 ± 12.4	
**Larynx** ( Volume [cm^3^] = 37.1 ± 19.9)				
Mean [%]	67.8 ± 3.7	69.0 ± 3.7	69.1 ± 3.7	a,b
D_1%_ [%]	93.1 ± 6.4	94.0 ± 7.5	92.8 ± 7.3	c
D_33%_ [%]	73.6 ± 4.2	74.8 ± 4.3	74.8 ± 4.3	a,b

a Acuros vs AAA, b Acuros vs CC, c AAA vs CC.

**Table 3:** **Thorax** (**Prescription 46**.**2 Gy** [**39**.**6**.-**50**.**0**])

**Parameter**	**Acuros XB**	**AAA**	**CC**	**p**
**PTV** ( Volume [cm^3^] = 516.8 ± 398.3)				
Mean [%]	100.3 ± 1.0	101.2 ± 0.4	100.9 ± 1.2	
D_5%_- D_95%_ [%]	10.3 ± 1.6	9.8 ± 3.0	10.8 ± 2.6	c
V_95%_ [%]	94.3 ± 2.2	95.3 ± 2.4	94.4 ± 3.3	a
V_105%_ [%]	4.1 ± 2.4	6.8 ± 5.7	7.0 ± 5.1	
CI_90%_	1.8 ± 1.2	1.9 ± 1.2	2.0 ± 1.1	
**Ipsil Lung** ( Volume [cm^3^] = 1700.2 ± 318.3)				
Mean [%]	43.3 ± 24.7	43.3 ± 24.7	42.6 ± 24.5	
V_20Gy_ [%]	48.0 ± 25.7	47.8 ± 25.7	47.5 ± 25.7	b
D_1%_ [%]	104.3 ± 0.9	103.9 ± 0.9	104.5 ± 0.2	a
**Contr Lung** ( Volume [cm^3^] = 2048.4 ± 42.1)				
Mean [%]	9.6 ± 2.6	9.8 ± 2.8	9.1 ± 2.3	
V_20Gy_ [%]	3.2 ± 2.6	3.2 ± 2.6	3.0 ± 2.4	
D_1%_ [%]	58.2 ± 19.3	58.8 ± 19.9	57.1 ± 18.2	
**Lungs** ( Volume [cm^3^] = 3382.7 ± 479.6)				
Mean [%]	24.5 ± 7.0	24.9 ± 7.2	23.9 ± 6.9	b,c
V_20Gy_ [%]	19.8 ± 7.7	19.9 ± 7.7	19.4 ± 7.7	b,c
D_1%_ [%]	98.4 ± 4.5	98.4 ± 4.3	98.2 ± 4.6	
**Heart** ( Volume [cm^3^] = 501.3 ± 31.4)				
Mean [%]	39.7 ± 27.0	40.4 ± 27.6	39.7 ± 27.3	c
D_1%_ [%]	101.9 ± 3.0	103.5 ± 3.0	102.2 ± 3.9	a
**Spinal Canal** ( Volume [cm^3^] = 31.9 ± 3.3)				
D_1%_ [%]	67.6 ± 23.3	68.5 ± 23.5	67.9 ± 23.1	a
D_1.8cm3_ [%]	63.7 ± 23.4	64.5 ± 23.5	64.1 ± 23.5	b

a Acuros vs AAA, b Acuros vs CC, c AAA vs CC.

**Table 4:** **Pelvis** (**Prescription 51**.**6 Gy** [**45**.**0**.-**56**.**0**])

**Parameter**	**Acuros XB**	**AAA**	**CC**	**p**
**PTV** ( Volume [cm^3^] = 817.6 ± 527.5)				
Mean [%]	98.5 ± 3.3	100.3 ± 3.3	98.9 ± 3.2	a,c
D_5%_- D_95%_ [%]	6.8 ± 0.9	6.3 ± 1.0	6.7 ± 1.2	a,c
V_95%_ [%]	80.3 ± 35.1	88.9 ± 22.2	82.5 ± 28.7	
V_105%_ [%]	1.1 ± 1.6	4.4 ± 5.6	1.8 ± 3.8	
CI_90%_	1.2 ± 0.1	1.3 ± 0.1	1.2 ± 0.1	a,c
**Bladder** ( Volume [cm^3^] = 259.3 ± 114.6)				
Mean [%]	70.4 ± 16.4	72.2 ± 16.8	70.6 ± 16.5	a,c
D_1%_ [%]	101.6 ± 2.5	103.6 ± 2.8	102.1 ± 2.8	a,c
D_67%_ [%]	55.3 ± 29.0	56.7 ± 29.7	55.2 ± 29.1	a,c
**Rectum** ( Volume [cm^3^] = 101.6 ± 44.8)				
Mean [%]	68.4 ± 17.3	70.0 ± 17.5	68.3 ± 16.9	a,c
D_1%_ [%]	100.4 ± 4.2	101.9 ± 4.0	100.6 ± 4.1	a,c
D_67%_ [%]	53.4 ± 28.1	55.0 ± 28.5	53.3 ± 27.9	a,c
**Femurs** ( Volume [cm^3^] = 172.7 ± 46.3)				
Mean [%]	38.9 ± 6.8	40.0 ± 7.0	38.7 ± 7.0	a,c
D_1%_ [%]	75.8 ± 17.9	77.9 ± 18.5	76.0 ± 17.9	a,c
D_1.8cm3_ [%]	75.7 ± 18.0	77.8 ± 18.6	75.9 ± 18.0	a,c
**Bowel** ( Volume [cm^3^] = 1062.2 ± 903.1)				
Mean [%]	28.0 ± 19.4	28.8 ± 19.6	27.3 ± 19.2	a,b,c
D_1%_ [%]	68.8 ± 45.9	69.9 ± 46.3	68.5 ± 45.9	a,c
D_1.8cm3_ [%]	69.0 ± 48.1	69.9 ± 48.4	68.5 ± 48.0	a,b,c

a Acuros vs AAA, b Acuros vs CC, c AAA vs CC.

**Table 5:** **SBRT** (**Prescription 60**.**0 Gy** [**60**.**0**.-**60**.**0**])

**Parameter**	**Acuros XB**	**AAA**	**CC**	**p**
**PTV** ( Volume [cm^3^] = 68.9 ± 50.2)				
Mean [%]	102.2 ± 2.4	102.9 ± 3.4	102.0 ± 2.7	
D_5%_- D_95%_ [%]	19.2 ± 4.0	18.1 ± 2.5	20.2 ± 3.6	b
V_95%_ [%]	91.9 ± 3.0	93.2 ± 4.0	91.0 ± 3.1	b,c
V_105%_ [%]	35.0 ± 16.9	41.2 ± 21.4	35.2 ± 17.0	
CI_90%_	1.1 ± 0.2	1.2 ± 0.2	1.1 ± 0.2	b,c
**Ipsil Lung** ( Volume [cm^3^] = 1202.5 ± 421.1)				
Mean [%]	14.7 ± 12.7	14.8 ± 12.6	14.8 ± 12.5	
V_20Gy_ [%]	15.1 ± 17.4	15.0 ± 17.3	15.3 ± 17.1	
D_1%_ [%]	74.8 ± 43.5	75.2 ± 43.8	74.9 ± 43.7	
**Contr Lung** ( Volume [cm^3^] = 1164.7 ± 601.0)				
Mean [%]	2.4 ± 2.0	2.4 ± 2.0	2.2 ± 1.9	
V_5Gy_ [%]	5.4 ± 10.5	5.1 ± 9.9	4.9 ± 9.4	
D_1%_ [%]	7.9 ± 4.9	8.0 ± 4.9	7.7 ± 5.0	b,c
**Ribs** ( Volume [cm^3^] = 213.5 ± 327.2)				
D_1%_ [%]	83.3 ± 15.0	85.1 ± 15.7	83.7 ± 14.7	a
V_30Gy_ [%]	19.0 ± 18.7	20.1 ± 19.9	19.1 ± 18.8	
**Liver** ( Volume [cm^3^] = 1135.0 ± 489.9)				
Mean [%]	14.7 ± 4.3	15.0 ± 4.3	14.5 ± 4.3	a,b,c
D_1%_ [%]	88.6 ± 33.1	90.1 ± 33.8	89.0 ± 33.3	
V_21Gy_ [%]	11.6 ± 6.2	11.8 ± 6.2	11.6 ± 6.1	

a Acuros vs AAA, b Acuros vs CC, c AAA vs CC.

### Dose calculation algorithms and experimental instrumentation

RapidArc plans were optimized using the Progressive Resolution Optimiser algorithm (PRO 10.0.28) [22] implemented in the Eclipse planning system (Varian Medical Systems, USA) and dose calculations were performed using for each case both the Anisotropic Analytical Algorithm (AAA) [23] and the Acuros-XB algorithm [24] (version 10.0.28 for both) using a spatial resolution of 2.5 mm in the x and y directions. All plans were optimized and calculated for 6MV photon beams generated by a Clinac iX equipped with a Millennium 120 Multileaf Collimator. Given the variability of the clinical cases, RA plans included full, partial, single and multiple arcs to cover the spectrum of routine application of the treatment technique.

The COMPASS system (IBA Dosimetry, Germany), in its version 2.0.7, was used to generate independent data for the verification of the accuracy of the two Eclipse algorithms with respect to actual delivery. For a detailed description of the COMPASS system, readers are referred to the original publications [17,18]. Its principle can be summarized as follows. A detector is mounted on the linac gantry (typically at the accessory mount holder) and it is used to measure the fluence produced by the linac for a given field (static or dynamic, modulated or plain). The measured fluence is used then as input data for a 3D convolution algorithm (Collapsed Cone) which allows to reconstruct the dose ‘’delivered” by the linac in a CT dataset (which could be a phantom or a patient set, even a Cone Beam CT). In the present case, the same CT sets were used for RA planning and for COMPASS calculations. The dose calculation was performed with a resolution of 2.5 mm. The detector used for fluence measurements is the Matrixx 2D array of ion chambers with a spatial resolution of 7.6 mm (center-to-center distance of the chambers). Fine interpolation of data to build an high resolution fluence is part of the COMPASS algorithm itself. In this study, the entire COMPASS system can be considered as a pre-treatment quality assurance tool (since it was used in absence of the patient) and it was used to benchmark the accuracy of the Eclipse calculations vs. recalculation from actual fluence delivery. In the present study no assessment of the intrinsic accuracy of CC is provided and readers are referred to Korrevaar et al [17] for an appraisal.

### Analysis and evaluation tools

To appraise the accuracy of the algorithms from Eclipse with respect of the calculations of COMPASS, three levels of tests were designed.

The first level of investigation was based on the conventional analysis of parameters derived from Dose Volume Histograms (DVH). To avoid possible biases in the construction of DVHs, the 3D dose matrices from COMPASS were imported in Eclipse so that only one engine was applied to build them. The analysis included for target volumes (PTV) the mean dose, the coverage expressed as the volume receiving 95% or 105% of the prescription dose (V_95%_ and V_105%_), the homogeneity expressed as the difference between the dose to 5% and to 95% of it (D_5%_-D_95%_) and the conformality expressed as the ratio between the volume of the 95% isodose and the PTV (conformity index, CI_95%_). For organs at risk, various parameters were quantified depending on the specificity of each of them in the spirit of ICRU 83 recommendations [25]. These included: mean doses, maximum significant doses (e.g. D_1%_ or D_1.8cm3_), doses to a given volume (D_x%_) and volumes receiving given dose levels (V_x%_).

The second level of investigation was aiming to quantify global differences in the dose distributions between the different algorithms and conditions. This was better expressed in terms of mean dose difference for each PTV or organ at risk (depending on the groups) for the couples AAA – Acuros, CC - AAA and CC – Acuros. Positive differences indicated a dose over-estimation of the first algorithm with respect to the second, and vice-versa. Objective was to identify and quantify possible systematic trends.

The third level of analysis was aiming to determine the possible relevance of observed discrepancies. The adopted tool was the 3D gamma analysis based on a generalization of the gamma of Low concept [26]. The computational methods here adopted has been described in Fogliata et al. [27]. The 3D gamma test was applied to each of the volumes listed above representing the clinically relevant objects. To appraise also the accuracy in the low dose range, the test was applied also to the volume of patients encompassed by the 50% and 10% isodoses. All tests were repeated using two sets of thresholds: a conventional dose difference and distance to agreement (DTA) of 3%/3 mm, used in routine clinical practice for quality assurance purposes, and a more restrictive 2%/2 mm aiming to stress the algorithms at the limit of their calculation resolution. Results were expressed in terms of pass rates, i.e. the percentage of voxels in a volume passing the gamma test. As a general concept, in the comparison between AAA (or Acuros) and COMPASS-CC, low pass rates would suggest the risk of relevant discrepancies between the dose delivered to a patient and the intended plan, outside the ‘’recovery” tolerances of the gamma measure. In the absence of any consensus on acceptability levels, it is assumed here that any pass rate higher than 97% corresponds to completely satisfactory agreement while, conversely, pass rates inferior to 90% would recommend some care suggesting possible clinical risks.

For all comparisons, statistical significance at 5% was assessed by means of Fisher’s signed test.

## Results

Tables 1, 2, 3, 4 and 5 present a summary of the quantitative comparison of DVH obtained from the dose distributions computed with Acuros-XB and AAA and from the CC based calculations on the experimental COMPASS. Data are presented separately for the four groups showing for each parameter the mean over the patients in the group and the standard deviation; in the p column are identified the cases where significant differences were observed. For each organ at risk or target volume it is reported also the mean volume and its standard deviation. All data are reported in % because of the different dose prescriptions. Although sometimes statistically significant, no macroscopic discrepancies were observed between all algorithm for all parameters.

**Figure 1:**
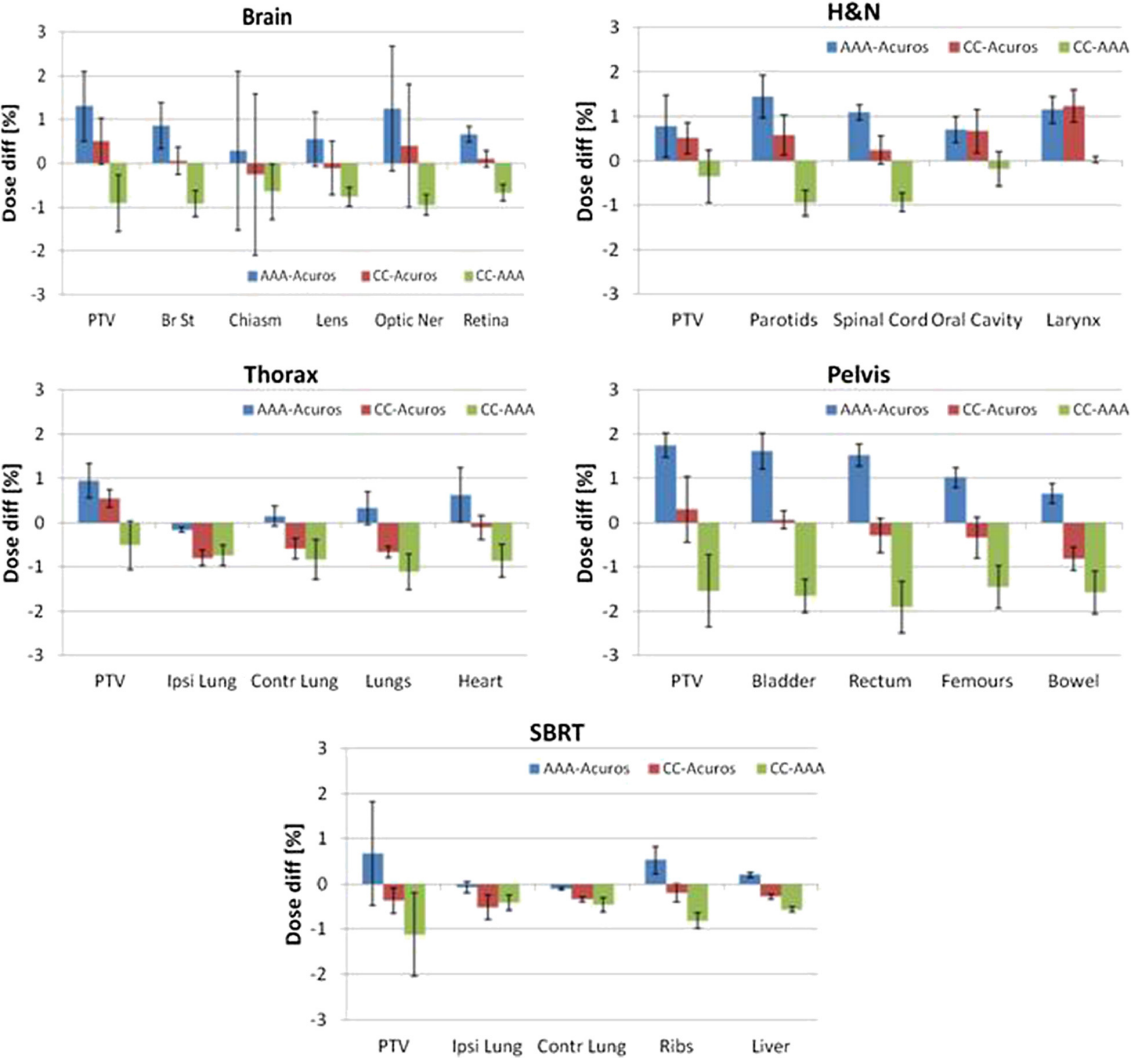
**Graphical summary of the average percentage dose difference between the various algorithms for various significant volumes or organs.** Data, obtained from 3D dose distributions, are presented separately for the five groups: brain, head and neck, thorax, pelvis and SBRT clinical cases.

Figure 1 contains the graphical summary of the average percentage dose difference between the three algorithms for the target volumes and organs at risk for the five groups. Data, obtained from 3D dose distributions of the plan differences, are presented separately for the five groups. The error bars represent one standard deviation. As a general trend, AAA over-estimate the dose compared to Acuros and to CC. Acuros and CC are on average in better agreement with a more variable pattern of over- and under- estimation of the doses. The average overestimation of AAA respect to Acuros for the analyzed organs is 0.70 ± 0.69% and of AAA respect to COMPASS-CC is 0.88 ± 0.46%. The average difference between Acuros and COMPASS-CC is −0.02 ± 0.51%. In all cases for brain, head and neck, thorax and SBRT, average differences did not exceeded 1.3%, for pelvis these reached 1.8%. In most of the cases the observed differences resulted statistically significant with the exception of the chiasm in the brain, the larynx in head and neck, the lungs in the thorax and the PTV and liver in SBRT, where no significance was determined.

**Figure 2:**
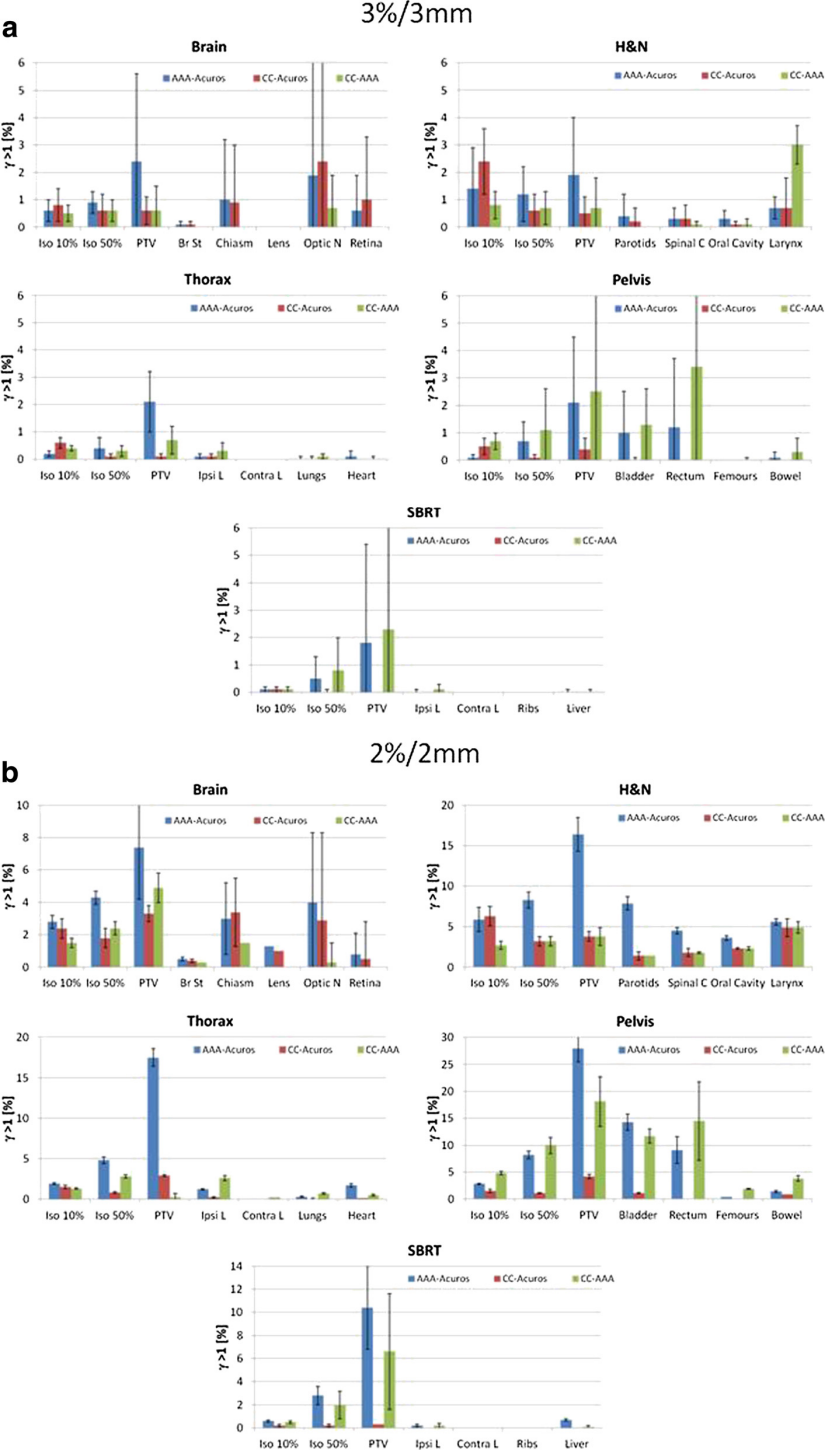
**Graphical summary of the failure rate after 3D gamma analysis (with thresholds set to 3%/3 mm (a) or to 2%/2 mm (b)).** Data, presented for various significant volumes or organs at risk, are presented separately for the five groups: head and neck, thorax, pelvis and SBRT clinical cases.

Figure 2 is the graphical summary of the 3D gamma analysis. Results are shown in terms of the residual failure rate expressed as percentage of voxels in the volumes under analysis not passing the gamma test with thresholds set to 3%/3 mm (a) or to 2%/2 mm (b). As for the previous results, data are presented for the five groups separately and for the same volumes of interest. The average pass rates (expressed as Gamma Agreement Index, i.e. the percentage of voxels in the organ passing the 3D gamma test) are summarized in Table 6. In general, with the conventional thresholds of 3%/3 mm, all algorithms did agree with a maximum failure of ~3% (larynx and rectum). With the tighter thresholds, Acuros and COMPASS-CC remained highly consistent with average failure rate inferior to ~3% with only one exception for the low isodose and larynx case in the head and neck (~6% and ~4% respectively). High failure rates were observed for AAA compared to Acuros or COMPASS-CC, particularly for PTVs. A general better agreement between AAA and COMPASS-CC was observed than between AAA and Acuros.

**Table 6:** Gamma agreement index

**Group**	**AAA**_**Acuros**	**CC**_**Acuros**	**CC**_**AAA**	**p**
**GAI [%] with criteria 3 %/3 mm**				
Brain	99.1 ± 2.1	99.2 ± 2.1	99.7 ± 0.6	b
H&N	99.0 ± 1.4	99.4 ± 0.9	99.4 ± 1.0	a,b
Thorax	99.6 ± 0.8	99.9 ± 0.2	99.7 ± 0.33	-
Pelvis	99.4 ± 1.4	99.9 ± 0.3	98.9 ± 3.1	a,c
SBRT	99.6 ± 1.4	100.0 ± 0.0	99.5 ± 2.1	-
**GAI [%] with criteria 2 %/2 mm**				
Brain	97.0 ± 4.5	98.0 ± 3.6	98.7 ± 3.4	a,b
H&N	91.1 ± 7.3	96.9 ± 3.2	96.2 ± 5.5	a,b
Thorax	96.4 ± 5.7	99.2 ± 1.3	97.9 ± 2.4	a,c
Pelvis	92.2 ± 11.3	98.8 ± 2.3	91.9 ± 14.4	a,c
SBRT	97.8 ± 4.8	99.9 ± 0.2	98.5 ± 4.9	a

a AAA_Acuros vs CC_ Acuros, b AAA_ Acuros vs CC_AAA, c CC_ Acuros vs CC_AAA.

## Discussion and conclusion

The aim of the present study was the assessment of the degree of agreement between 3D dose distributions calculated for clinical RA plans against independent calculations based on actual fluence delivered by the linear accelerators, before entering in the patient. These fluences were used to calculate the dose ‘delivered’ to the patients using the planning CT dataset. In this way, the object of the study is in practice the appraisal of the accuracy of the planning calculation engines in simulating the real delivery by the linear accelerator. In fact, errors and issues attributable to changes in patient position, anatomy and shape are not accounted for because all calculations were performed on the same planning CT, a single snapshot in time. The study as presented here, cannot provide an absolute determination of the accuracy of the clinical algorithms because, in the experimental arm, another algorithm (CC) is used by the COMPASS system. This kind of loop is unavoidable (and present also in the Monte Carlo based methods or in the EPID based [16] methods) because whatever the strategy, it is always necessary to convert some kind of measurement into a 3D dose distribution inside the patient. Validation of the CC algorithm was not subject of this study and was addressed by its developers in their founding studies. Here CC accuracy was considered to be adequate for quality assurance purpose as determined by Korevaar [17] or Nakaguchi [19] and comparable to what achievable with films or other dosimetry devices (Mapcheck). Within the frame of validity defined above, the two algorithms available for clinical calculation of RA plans, Acuros-XB and AAA were compared against benchmark data from COMPASS for a total of 25 patients, divided in 5 groups representing different treatment sites, dose prescription and plan complexity. From the three different analyses performed on the data it is possible to extract some general consideration. Based on DVH analysis, both Acuros-XB and AAA resulted in good agreement with COMPASS-CC calculations. Acuros-XB showed smaller differences than AAA for basically all parameters usually used for plan evaluation and for dose reporting as recommended by ICRU [25]. This fact is reassuring because it suggests that, for RA, the calculation engines, with all their inherent approximations, are anyway adequate to model the real delivery within acceptable levels. In fact the differences reported in Tables 1, 2, 3, 4 and 5 would not be considered clinically alarming and could be well ascribed to the intrinsic variation between different algorithms. In this respect it is important to notice that for most of the patients, the anatomical sites studied included highly heterogeneous tissues which are differently managed and modeled by the different algorithms as demonstrated in earlier studies [28-30]. The same results suggest also that even if 3D dose reconstruction in patients are available as part of advanced quality assurance procedures, an analysis based only on DVH parameters could be insufficient to determine possibly clinical relevant features. More interesting results were in fact obtained from the inspection of 3D dose differences per organ. In this case, it was possible to demonstrate the systematic difference between Acuros-XB and AAA with respect to COMPASS-CC and the smaller discrepancies when Acuros-XB is used. In addition, it was possible to demonstrate that calculations based on AAA have a systematic trend of over-estimation of the dose actually delivered to the patients. Although the absolute values are small (below 2%), this could have some clinical impact (e.g. with AAA more plans might be considered not acceptable then Acuros-XB due to dosimetric constraints violations). Finally, the application of more complex tools like the 3D gamma, allowed to determine that Acuros-XB is more robust and accurate than AAA with also tight thresholds (2%/2 mm). The clinical relevance of this relies on the fact that, in the absence of any perturbation due to patient positioning or organs motion, Acuros-XB reproduces almost perfectly the delivery suggesting its lower sensitivity to the two above elements if compared to AAA.

In conclusion, this study demonstrated that i) a good agreement exists between COMPASS-CC calculations based on measured fluences with respect to dose distributions obtained with both Acuros-XB and AAA algorithms; ii) 3D dose distributions reconstructed from actual delivery coincide very precisely with the planned data; iii) a slight preference in favor of Acuros-XB was observed suggesting the preference for this algorithm in clinical applications.

## Competing interests

The corresponding author states: Dr. L. Cozzi acts as a scientific advisor to Varian Medical Systems and is Head of Research and Technological Development to Oncology Institute of Southern Switzerland, IOSI, Bellinzona.

## Authors’ contributions

VSS and LC coordinated the entire study. Data collection was conducted by MK. VSS, VS, ST. Analysis tools were developed and data processing was done by AFC, GN, EV, AC. LC wrote the manuscript. All authors reviewed and approved the final version.
